# Pulmonary Manifestations of Inflammatory Bowel Disease in Children

**DOI:** 10.7759/cureus.11369

**Published:** 2020-11-07

**Authors:** Mark A Taylor, Holly Zhou, David M Dansie, Scott Short

**Affiliations:** 1 Department of Surgery, University of Utah, Salt Lake City, USA; 2 Department of Pathology, University of Utah, Salt Lake City, USA; 3 Department of Radiology, Primary Children's Hospital, Salt Lake City, USA

**Keywords:** lung, ulcerative colitis, crohn's, nodule

## Abstract

The recognition of pulmonary manifestations of inflammatory bowel disease (IBD) is important as these diseases can be confused with infectious etiologies (e.g., tuberculosis or fungal infection) and, as a result, may unnecessarily delay institution of appropriate therapy (e.g., infliximab). Furthermore, they are a source of morbidity that may be overlooked and, like other IBD-related pathologies, are often responsive to treatment with corticosteroids, immunomodulators, or biologic therapies. The purpose of this paper is to describe the cases of six children at a single institution with differing presentations, treatments, and responses to treatment of their IBD-related lung disease to improve recognition of this uncommon process.

## Introduction

Pulmonary manifestations are increasingly being recognized as complications of inflammatory bowel disease (IBD) since it was first described in 1976 [1,2]. Our series highlights six cases where surgical biopsy was required to definitively exclude infectious pulmonary processes prior to initiation of therapy.

## Case presentation

Case 1

**Figure 1:**
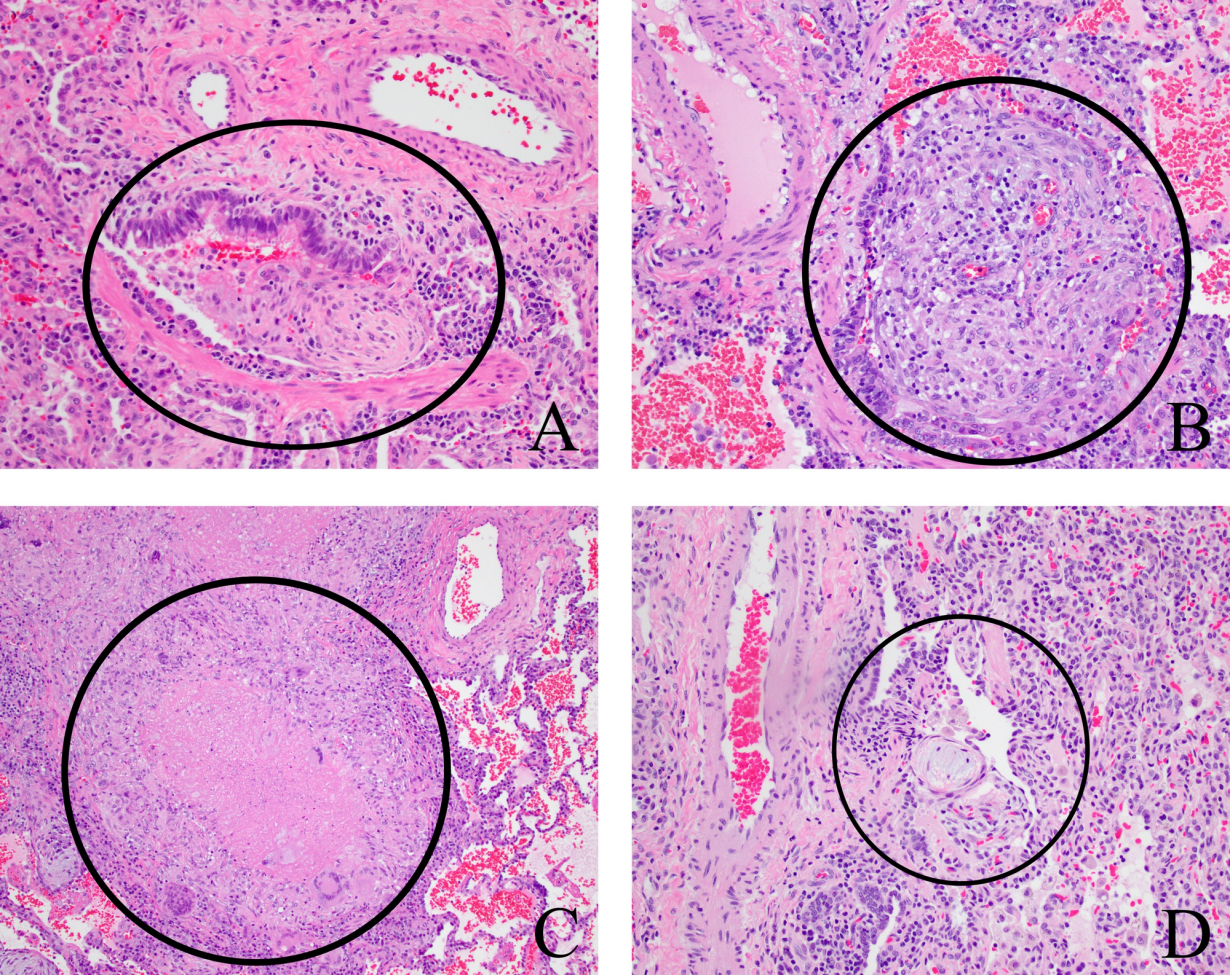
Representative histologic findings of patients with pulmonary manifestations of inflammatory bowel disease. All images are H&E stain. Pathology indicated by black circles. A: Case 1 demonstrating cryptogenic organizing pneumonia; B: Case 2 demonstrating a non-necrotizing granuloma centered on a bronchiole; C: Case 3 demonstrating necrotizing granulomatous bronchiolitis; D: Case 5 demonstrating focal organizing pneumonia. H&E, hematoxylin and eosin.

A 16-year-old boy with a history of ulcerative colitis (UC), diagnosed at age 15, and complicated by primary sclerosing cholangitis presented with pleuritic chest pain and low-grade fevers in the setting of an IBD flare with presenting symptoms of six to seven bloody stools per day and abdominal pain with labs significant for a leukocytosis and elevated ESR and CRP. Prior to admission, his symptoms had been well controlled with azathioprine for maintenance therapy. A chest x-ray demonstrated right upper and left lower lobe pulmonary opacities, and a subsequent CT scan revealed multiple pulmonary nodules with associated hilar lymphadenopathy. Due to concern regarding a possible infectious etiology that would preclude administration of additional medical therapy, a thoracoscopic biopsy was performed. There were no perioperative complications. The chest tube, which was placed routinely for the treatment of potential pulmonary air leak, was removed on post-operative day two after no air leak was identified. The pathology demonstrated necrotic neutrophilic nodules without granulomas, which were consistent with pulmonary manifestations of his UC. Operative cultures and additional infectious workup were negative. He was then treated with a three-month course of corticosteroids and started on mesalamine for maintenance therapy with subsequent improvement in his symptoms and a decrease in size of the nodules on CT scan 10 days after initiation of therapy. He presented again two years later with similar symptomatology in the setting of an IBD flare, and the nodules were noted to be larger, but in the same locations. Between these presentations, his UC maintenance therapy was changed to azathioprine. He was again treated with a three-month course of corticosteroids while continuing azathioprine with resolution of his symptoms and a decrease in size of the nodules on CT scan one month later. One year later, he had developed left-sided pleuritic chest pain and worsening of his UC symptoms. The decision was made to initiate infliximab for the management of his UC, and a CT scan was repeated to evaluate the nodules prior to change in therapy. The nodules were noted, again, to be larger, but in the same locations. Another thoracoscopic biopsy was performed to rule out an infectious etiology, which showed cryptogenic organizing pneumonia (COP; formerly called bronchiolitis obliterans organizing pneumonia), suppurative pneumonia, and macrophagic pneumonitis, again, favored to represent pulmonary manifestations of IBD (Figure 1A). This was, again, suggestive of pulmonary manifestations of his UC. Infliximab was initiated, and symptoms dramatically improved, with resolution of nodules noted on CT scan four months later.

Case 2

**Figure 2:**
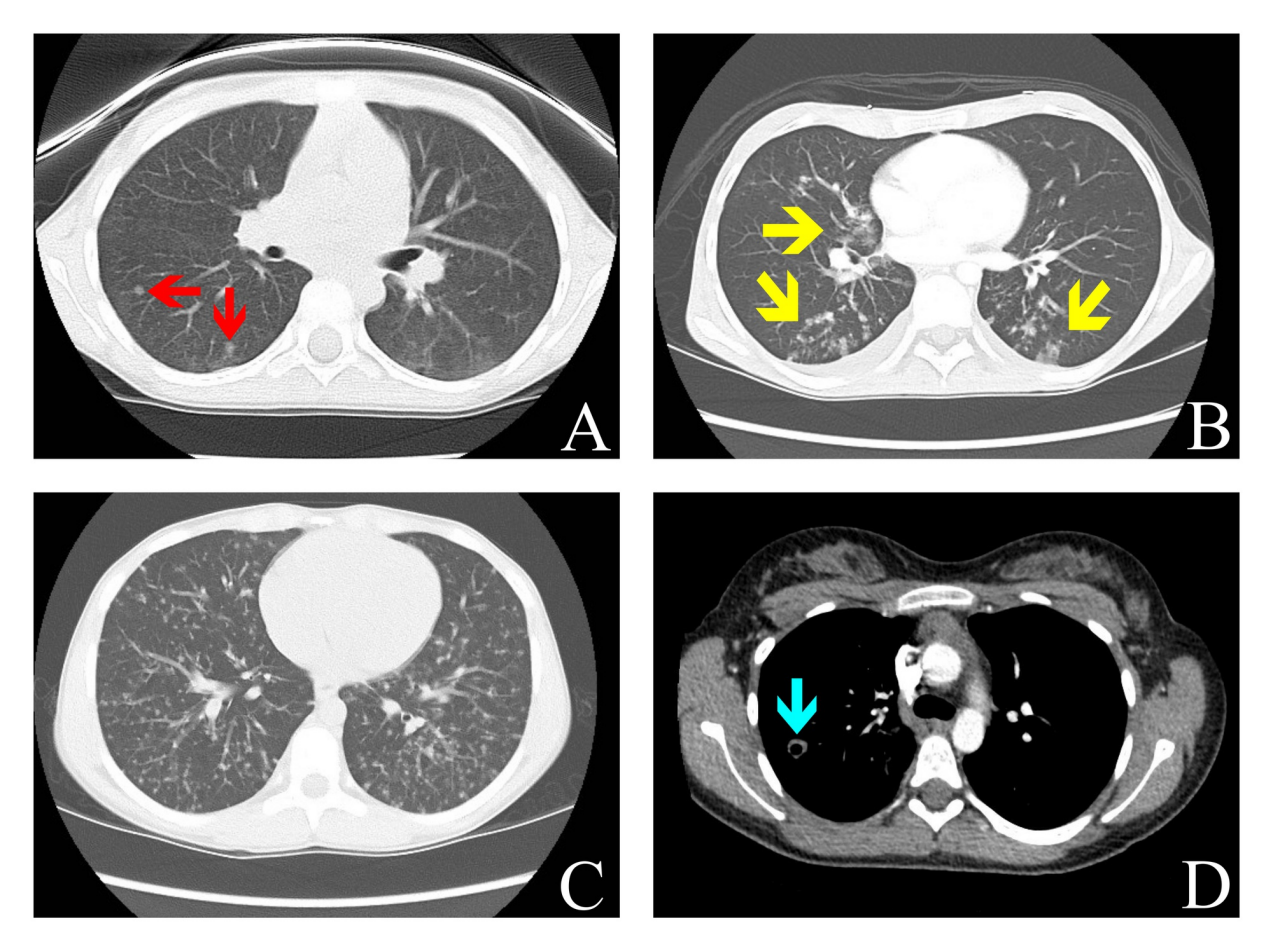
Representative computed tomography (CT) images of patients with pulmonary manifestations of inflammatory bowel disease. A: Case 2 with two representative nodules in the right lung (red arrows); B: Case 3 with extensive, bilateral tree-in-bud opacities (yellow arrows); C: Case 4 with diffuse pulmonary micronodules; D: Case 5 with nodule with central cavitation in the right lung (blue arrow).

A 10-year-old girl with a history of Crohn’s disease, diagnosed at age seven, presented with an IBD flare and was incidentally found to have multiple small bilateral pulmonary nodules on CT scan obtained to investigate her abdominal pain (Figure 2A). Her Crohn’s disease was being managed with adalimumab at the time of presentation after previous failure to maintain remission on azathioprine, methotrexate, and infliximab. To rule out an infectious etiology prior to increasing her azathioprine, a thoracoscopic biopsy was performed after marking by interventional radiology (IR), which demonstrated non-necrotizing granulomatous bronchiolitis thought to be secondary to her Crohn’s disease (Figure 1B). Her perioperative course was uncomplicated. Her chest tube, which was placed routinely for the treatment of potential pulmonary air leak, was removed on post-operative day one after no air leak was identified. Further infectious workup was negative. Her adalimumab dose was increased, and she remained asymptomatic. From a pulmonary standpoint the pulmonary nodules had decreased in size one year after biopsy, as identified on repeat CT scan obtained to reevaluate the nodules.

Case 3

A 17-year-old girl presented to the surgical clinic with profound malnutrition with an albumin of 1.3 and BMI of 12.9 for evaluation for gastrostomy tube placement. Her medical history was notable for a prior drainage of perirectal abscesses and behavioral evaluations for anorexia nervosa. In addition, she had a two-year history of watery diarrhea about three times per day. On clinical exam, she was noted to have complicated and extensive perianal abscesses and fistulae. Due to her severe malnutritive state, she was admitted to the hospital for total parenteral nutrition, management of her refeeding syndrome, and work-up for IBD. Colonoscopic biopsies revealed Crohn’s disease. During her workup, a CT of her abdomen demonstrated opacities in the bilateral lower lobes of the lung, and a subsequent CT chest demonstrated extensive tree-in-bud opacities predominantly in the bilateral lower lobes of the lung (Figure 2B). Due to the unclear etiology of these pulmonary lesions and the need to start therapy for her Crohn’s disease, a biopsy of these lung lesions was recommended. However, given her significant malnutrition and asymptomatic pulmonary disease, the decision was made to improve her nutritional status prior to surgical biopsy. After a month of nutrition, a 20-pound weight gain, and an improvement in her albumin to 3.4, a thoracoscopic biopsy was performed and demonstrated necrotizing granulomatous inflammation and focal organizing pneumonia favored to represent pulmonary manifestations of IBD (Figure 1C). Infectious workup was negative. Her perioperative course was uncomplicated. Her chest tube, which was placed routinely for the treatment of potential pulmonary air leak, was removed on post-operative day one after no air leak was identified. A repeat CT scan was performed just prior to the biopsy (one month after the initial CT scan) to assist with operative planning. This demonstrated persistent, but significantly improved tree-in-bud opacities in the bilateral lower lobes without any treatment with steroids or anti-microbials and prior to initiation of any immunotherapy for her Crohn’s disease. She was subsequently started on infliximab without any repeat imaging of her chest.

Case 4

A 14-year-old boy presented with a nonproductive cough for one week after accidentally aspirating river water. He was diagnosed one month prior with Crohn’s disease after presenting with abdominal pain, diarrhea, and aphthous stomatitis and was being treated with a course of prednisone for this flare with improvement in his gastrointestinal symptoms. During the workup for his cough, he was found to have diffuse pulmonary micronodules on CT scan (Figure 2C). His cough resolved shortly after presentation without specific intervention. A thoracoscopic biopsy was performed to rule out an infectious etiology prior to starting maintenance therapy for his Crohn’s disease, which demonstrated granulomatous inflammation secondary to pulmonary manifestations of IBD. His post-operative course was uncomplicated. His chest tube, which was placed routinely for the treatment of potential pulmonary air leak, was removed on post-operative day one after no air leak was identified. He was started on infliximab and methotrexate two weeks after initial presentation after infectious work-up returned negative. In addition, he was continued on a prednisone taper for a total three-month course. A repeat CT scan one year later, at which time he remained asymptomatic, showed marked regression of the innumerable pulmonary nodules during which time he remained on infliximab and methotrexate.

Case 5

A 14-year-old girl diagnosed at age 13 with Crohn’s disease was admitted with pleuritic chest pain and a non-productive cough. Her Crohn’s disease was controlled with infliximab, but this was discontinued due to the development of Henoch-Schönlein purpura (HSP) three months prior to this presentation. She was treated for the HSP with a prednisone taper that ended about a month prior to presentation and remained off maintenance therapy at the time of presentation. Due to persistent pulmonary symptoms, a chest x-ray was performed, which showed a 1.3cm right upper lobe nodule. A subsequent CT scan demonstrated bilateral pulmonary nodules, some of which demonstrate central cavitation, which raised concern for an infectious etiology, such as tuberculosis (Figure 2D). Thoracoscopic biopsy was performed after marking by IR to rule out an infectious etiology prior to treating with immunosuppressive medication. Her post-operative course was uncomplicated. Her chest tube, which was placed routinely for the treatment of potential pulmonary air leak, was removed on post-operative day three after no air leak was identified. The pathology demonstrated chronic interstitial pneumonitis with minimal patchy organizing pneumonia without granulomas favored to represent pulmonary manifestations of IBD (Figure 1D). Infectious workup was negative. Her pulmonary symptoms improved over the course of admission without any specific treatment. She was started on adalimumab two months after presentation for control of her Crohn’s disease, which was changed to vedolizumab three months later due to continued vasculitis on adalimumab. She had a repeat chest CT scan eight months after her initial CT scan to reevaluate the pulmonary nodules, which showed resolution of the previous pulmonary nodules with two new cavitary nodules in the right middle lobe and lingula.

Case 6

A 13-year-old girl diagnosed one year prior with UC presented with a chronic, productive cough and exertional dyspnea for one month. Her UC has been refractory to corticosteroids, infliximab, and vedolizumab, and she was having worsening bloody diarrhea complicated by anemia. She was currently not receiving any therapy and was discussing surgical options given her refractory disease. A chest x-ray was obtained for her pulmonary symptoms, which showed multiple pulmonary nodules. A subsequent chest CT scan demonstrated similar findings in addition to concern for organizing pneumonia. She was started on a seven-day course of ceftriaxone for the possible pneumonia, and, given the diagnostic uncertainty of these nodules, a CT-guided IR biopsy of a pleural-based nodule was obtained. Pathology demonstrated a fibroinflammatory process favoring cryptogenic organizing pneumonia without granulomas likely secondary to IBD. Further infectious workup was negative. After biopsy, given her refractory disease and ongoing anemia, she underwent a laparoscopic subtotal colectomy with end ileostomy. Her post-operative course was uncomplicated. Her pulmonary symptoms improved during her hospitalization, but she still had an occasional cough and exertional dyspnea. Pulmonary function testing (PFT) demonstrated evidence of mild persistent asthma with an FEV1 of 88% predicted, FVC of 95% predicted, and FEV1/FVC of 83% with improvement in the FEV1 by 15% after bronchodilator administration. She was started on albuterol as needed and a fluticasone inhaler. Repeat CT scan two months after the initial CT scan revealed significant improvement in the pulmonary nodules bilaterally and no evidence of residual pneumonia. At the time of this CT scan, her cough had resolved, but she had persistent dyspnea on exertion. Repeat PFT around the time of this CT scan revealed persistent asthma, but with a significant response to bronchodilator.

## Discussion

The incidence of pulmonary manifestations is unknown as many patients have subclinical disease, have symptoms attributed to other etiologies, or have symptoms unrecognized by the treating provider [3-5]. The etiology of these pulmonary manifestations is uncertain and, although multifactorial, may be related to the underlying embryologic origins of the pulmonary and gastrointestinal epithelia. The tissues of both the pulmonary and intestinal epithelia are derived from the foregut portion of the endoderm and, thus, share similar biologic features. The same factors driving the overexuberant immunologic response of the gastrointestinal (GI) tract are hypothesized to play a role in the development of pulmonary manifestations that occur in some patients [6]. Other proposed mechanisms include generalized systemic inflammation and microbial dysbiosis [4]. It is well known that IBD is characterized by systemic inflammation, and this inflammation contributes to priming of neutrophils, which, in turn, makes them easier to activate. In a normal physiologic state, the lung has been shown to play an important role in the de-priming of neutrophils as a means of maintaining immunologic homeostasis. However, in IBD, it has been shown that the de-priming role of the lung is impaired allowing for exuberant neutrophil activation with subsequent damage to the lung tissue [4]. In addition, patients with IBD lose the symbiotic relationship with the commensal bacteria in the gut, which drives many of the symptoms of IBD and affects the microbial composition of other mucosal surfaces including in the lungs. As a result of this microbial dysbiosis in the lungs, the activated neutrophils are theorized to attack the lung tissue, leading to the development of pulmonary manifestations of IBD [4].

Clinically, the pulmonary manifestations of IBD can occur in every part of the pulmonary system. In our series, we reported pleuritic chest pain in two patients, productive cough in four, and asymptomatic pulmonary pathology in two. Although, these presentations have been reported in other series, it appears that bronchiectasis and chronic bronchitis are more common in the adult populous [3]. Typically, adults have presented with large airway disease symptoms manifesting as inflammation of the trachea and bronchi with subsequent coughing, stridor, hoarseness, or dyspnea [3]. Our series, on the other hand, demonstrated parenchymal and, in some cases, pleural involvement in all children with pulmonary manifestations. Clinically, disease in these parts of the airway may present as a chronic, productive cough, pleural thickening, effusion, and/or pleuritis [2,3]. Pathologically, these manifestations are often identified as COP, as seen in patients 1, 5, and 6 and represent the most common pathologic finding seen in the pulmonary parenchyma [3,7]. Other pathologies reported include necrobiotic nodules (sterile accumulations of neutrophils in an area of necrosis) or granulomatous inflammation as seen in patients 1, 2, 3, and 4 [3,8]. Others have reported usual interstitial pneumonia, lymphocytic interstitial pneumonia, desquamative interstitial pneumonia, and eosinophilic interstitial pneumonia [2,3,4]. Radiographically and as seen in most of the patients in our series, these parenchymal and pleural pathologies can manifest as pulmonary nodules. Other radiographic findings include tracheal/bronchial thickening in larger airway disease and irregular linear opacities, ground glass opacities, interlobular septal thickening, and pleural thickening in parenchymal/pleural disease [3].

Standard treatment of pulmonary manifestations of IBD is not well described. As standard treatment for the gastrointestinal symptoms of IBD, most patients will receive immunosuppressant, immunomodulatory, or biologic therapy. However, it is necessary to exclude tuberculosis or other pulmonary infectious etiologies prior to instituting the therapy needed for treatment [9]. It is well known that tuberculosis can be activated with the institution of anti-TNF therapy with 1-2% of patients developing active tuberculosis after initiation of anti-TNF therapy prior to development of screening guidelines for latent TB in one study [10]. Although these therapies may treat the pulmonary manifestations of IBD, they also present another potential etiology of pulmonary disease that is necessary to exclude. Drug-related lung injury has been reported with use of salicylates, azathioprine, 6-MP, and methotrexate [2,3]. To further cloud the clinical picture, many of the associated drug toxicities can have a similar appearance on pathology to IBD-related pulmonary manifestations [3]. Because of this, there should be a low threshold to discontinue offending agents if drug-related lung injury is in the differential. Once infectious and drug-related causes have been ruled out, treatment should be considered. Often, the therapy utilized for the GI manifestations suffices, but the generally accepted first-line treatment for pulmonary manifestations is corticosteroids [2,3]. These can be delivered via inhalation or systemically, and the preferred route of administration depends on the pattern of disease. Airway disease is more likely to respond to inhaled corticosteroids, whereas parenchymal disease typically responds best to systemically administered corticosteroids [2]. Two of the patients in our series were treated with corticosteroids with partial resolution of the pulmonary disease. Another option for treatment either primarily or for refractory disease is immunomodulating and/or biologic medications [2,3,5]. All patients (patients 1, 2, 4, and 5) in our series treated with these medications for their IBD-related lung disease responded, at least partially, to this treatment. All had resolution of clinical symptoms if present. Interestingly, there are few reports on the effect colectomy for UC has on pulmonary manifestations. Patient six in our series had significant improvement in her symptoms and pulmonary nodules after removal of the diseased colon; however, there are variable reports in the literature on the effect of colectomy on the pulmonary manifestations of IBD [3,11,12].

## Conclusions

The diagnosis of IBD-related pulmonary manifestations should be considered in patients with IBD, who are either symptomatic or asymptomatic and found to have pulmonary disease on imaging incidentally. Drug-related injury and infection should be ruled out prior to treating with a drug aimed at decreasing the underlying systemic inflammation, such as corticosteroids, immunomodulators, or biologics. Symptoms and radiologic findings improve in the majority of patients with the standard treatment of IBD.
